# Supplementary material for: Time-resolved microfluidics unravels individual cellular fates during double-strand break repair
Source: BMC Biol. 2022 Dec 5;20:269. doi: 10.1186/s12915-022-01456-3 (PMC9720956; doi:10.1186/s12915-022-01456-3)
Supplement: Supplementary file 1 — Additional movie: Time-lapse microscopy of microfluidic wells. Table S1: RMSE of experimental population vs simulated data on Fig. 4. Figure S1: Southern blot quantification of DSB and DSBR for all experimental conditions. Figure S2: Test of Bayesian inference on simulated data with correctly specified model. Figure S3: Test of Bayesian inference on simulated data with non-Gaussian errors. Figure S4: Prior and posterior distributions of $\beta$β, $\rho$ρ, $\tau$τ and $\alpha$α for all experimental conditions. Figure S5: Posterior distributions of breaking ($\beta$β) and repair ( [file 12915_2022_1456_MOESM1_ESM.zip › AdditionalFile1.pdf]

# 9 Additional File 1

**Additional Movie: Time-lapse microscopy of microfluidic wells** 24 h time-lapse microscopy of random microfluidic wells with one single cell trapped at t0 for the 8 experimental conditions: NR, CGG, GAA or CTG target sequences with either Cas9 or Cpf1. Scale bar represents 50 $\mu$m.

| **Combination** | **RMSE** |
|---|---|
| NR - Cas9 | 0.022 |
| NR - Cpf1 | 0.160 |
| CGG - Cas9 | 0.055 |
| CGG - Cpf1 | 0.041 |
| GAA - Cas9 | 0.054 |
| GAA - Cpf1 | 0.082 |
| CTG - Cas9 | 0.093 |
| CTG - Cpf1 | 0.012 |

Table S1: **RMSE of experimental population vs simulated data on Figure 4**. A larger value of the RMSE indicates a poorer fit between experiments and simulations. The error is below 10% for all cases except two.

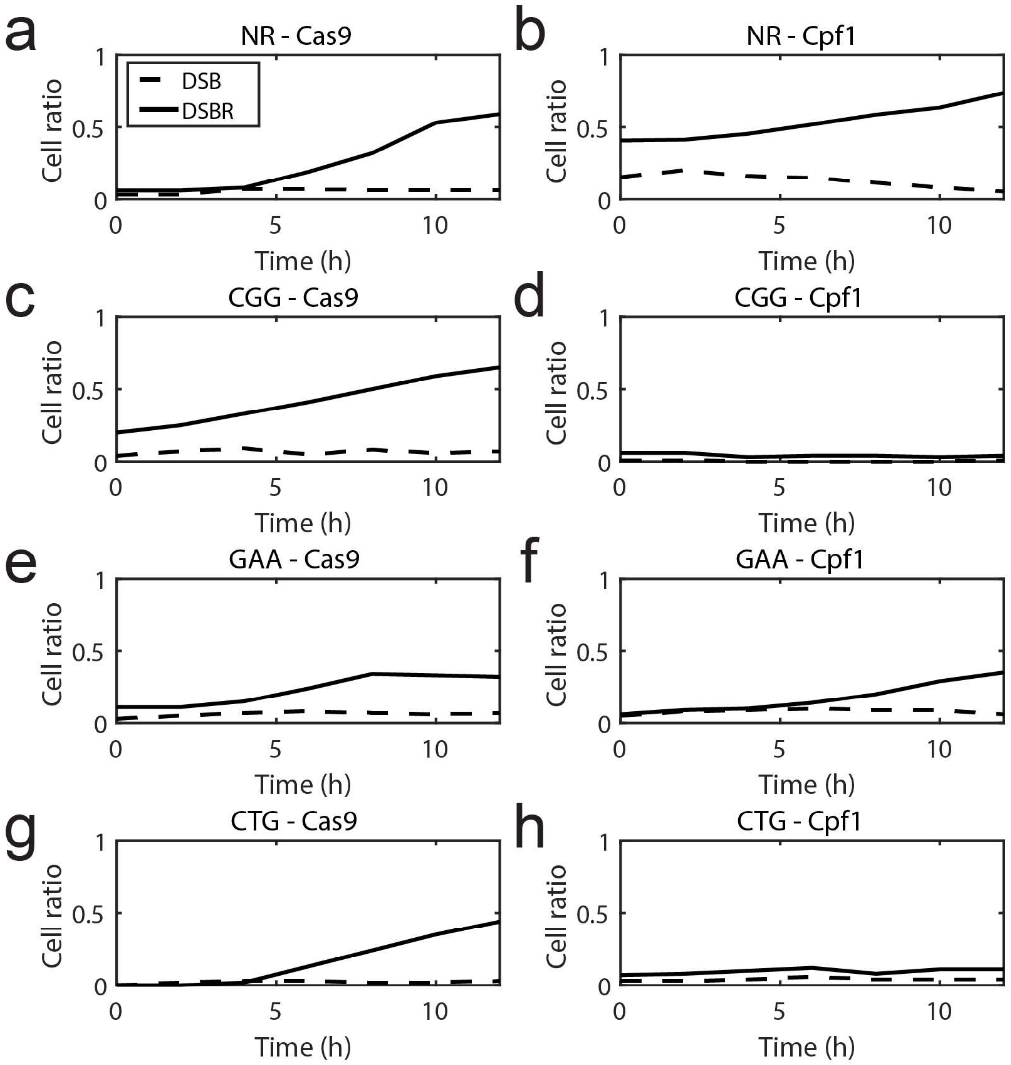


Figure S1: **Southern blot quantification of DSB and DSBR for all experimental conditions.** **(a)** NR - Cas9. **(b)** NR - Cpf1. **(c)** CGG - Cas9. **(d)** CGG - Cpf1. **(e)** GAA - Cas9. **(f)** GAA - Cpf1. **(g)** CTG - Cas9. **(h)** CTG - Cpf1. In all cases, dotted lines represent the fraction of cells which present broken chromosones (after DSB) and solid lines represent cells with the *GFP* gene (after completed DSBR). Adapted from Poggi *et al.* (12)

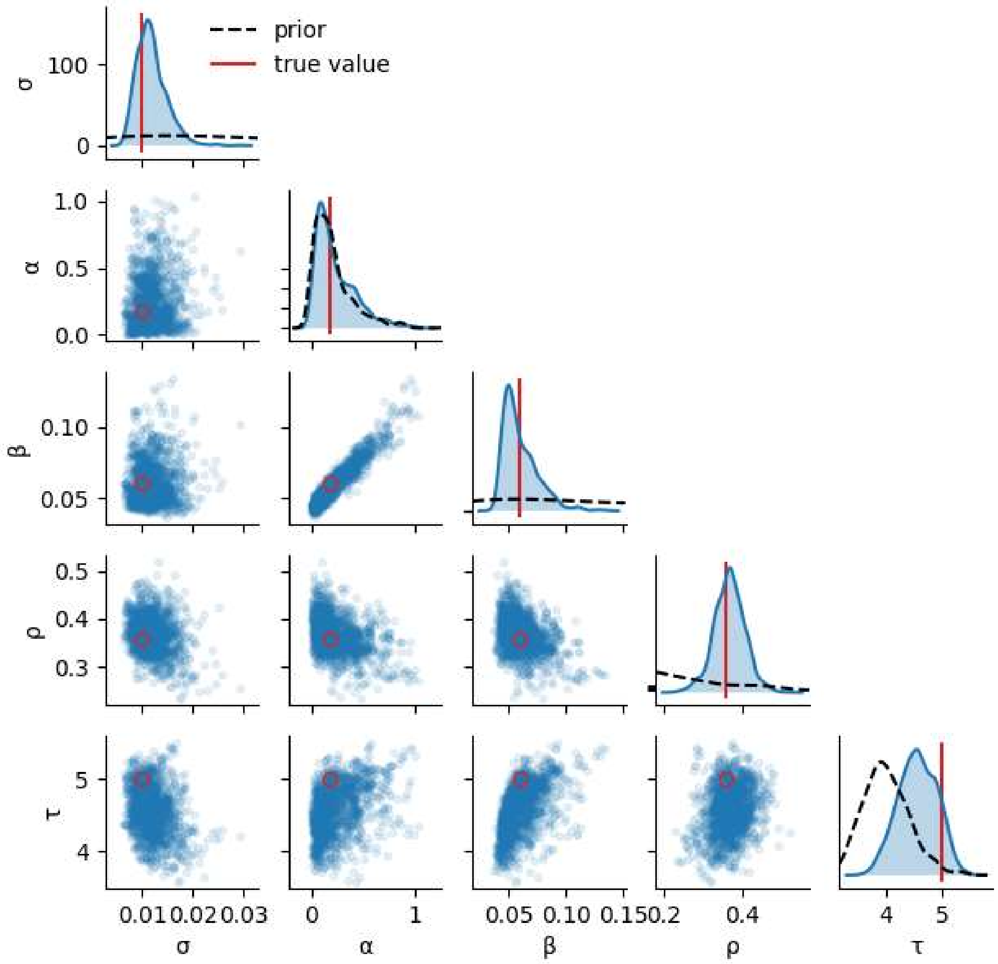


Figure S2: **Test of Bayesian inference on simulated data with correctly specified model.** Each plot on the diagonal shows a histogram of the posterior distribution (blue) along the with prior (dashed line) and the true parameter value used to generate the simulated data (red line). The plots below the diagonal show samples from the posterior distribution for different pairs of parameters, along the the true parameter values (red circle).

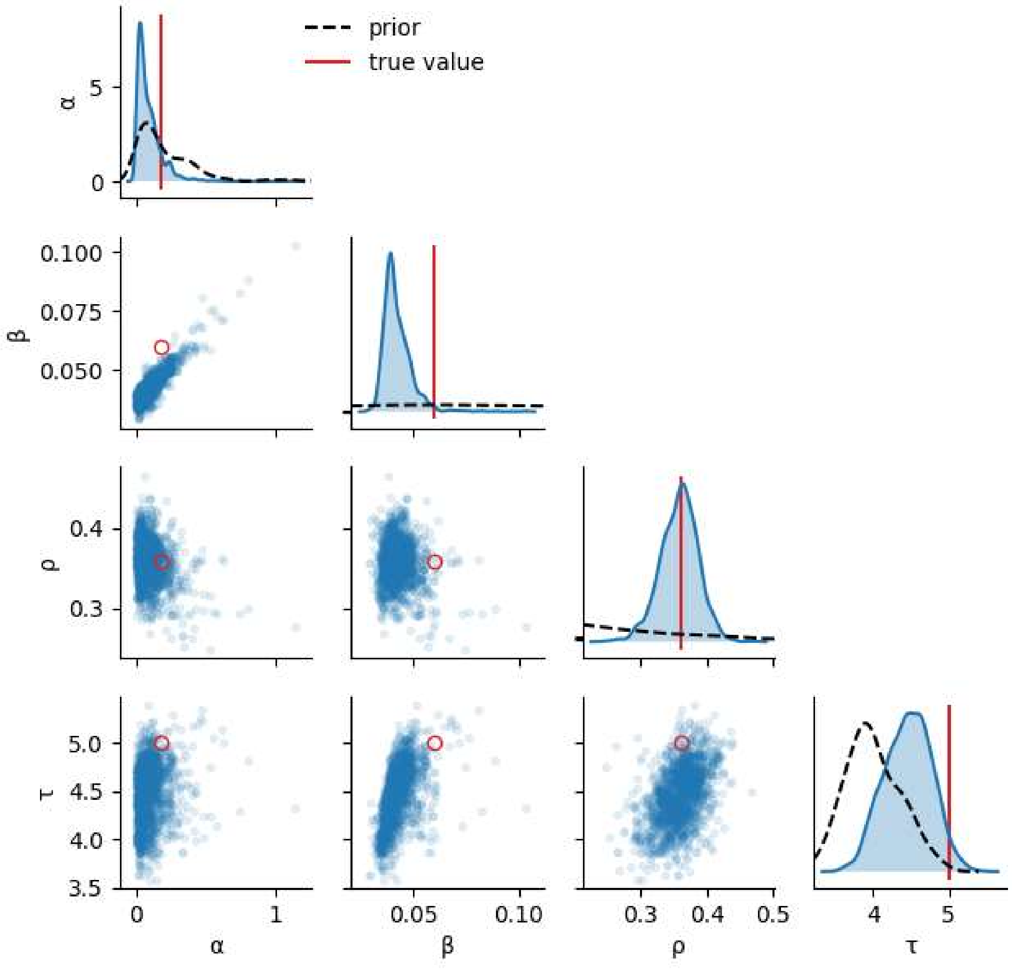


Figure S3: **Test of Bayesian inference on simulated data with non-Gaussian errors.** The same as Additional Figure S2, but using simulations in which the measurement error is not Gaussian. In particular, in this case we add measurement error to the absolute cell counts, which causes correlations in the noise between different cell types.

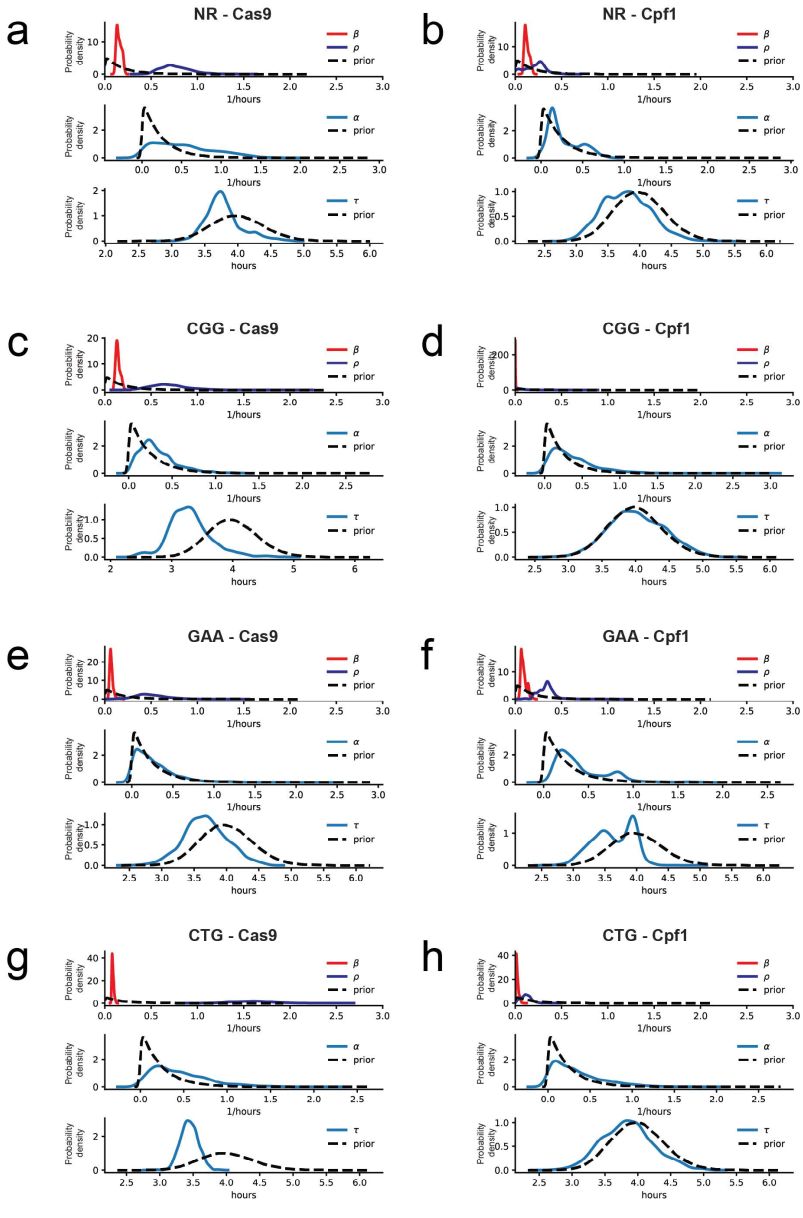


Figure S4: **Prior and posterior distributions of $\beta$, $\rho$, $\tau$ and $\alpha$ for all experimental conditions.** **(a)** NR - Cas9. **(b)** NR - Cpf1. **(c)** CGG - Cas9. **(d)** CGG - Cpf1. **(e)** GAA - Cas9. **(f)** GAA - Cpf1. **(g)** CTG - Cas9. **(h)** CTG - Cpf1. In all cases, dotted lines represent the prior distributions and solid lines represent the posterior distributions.

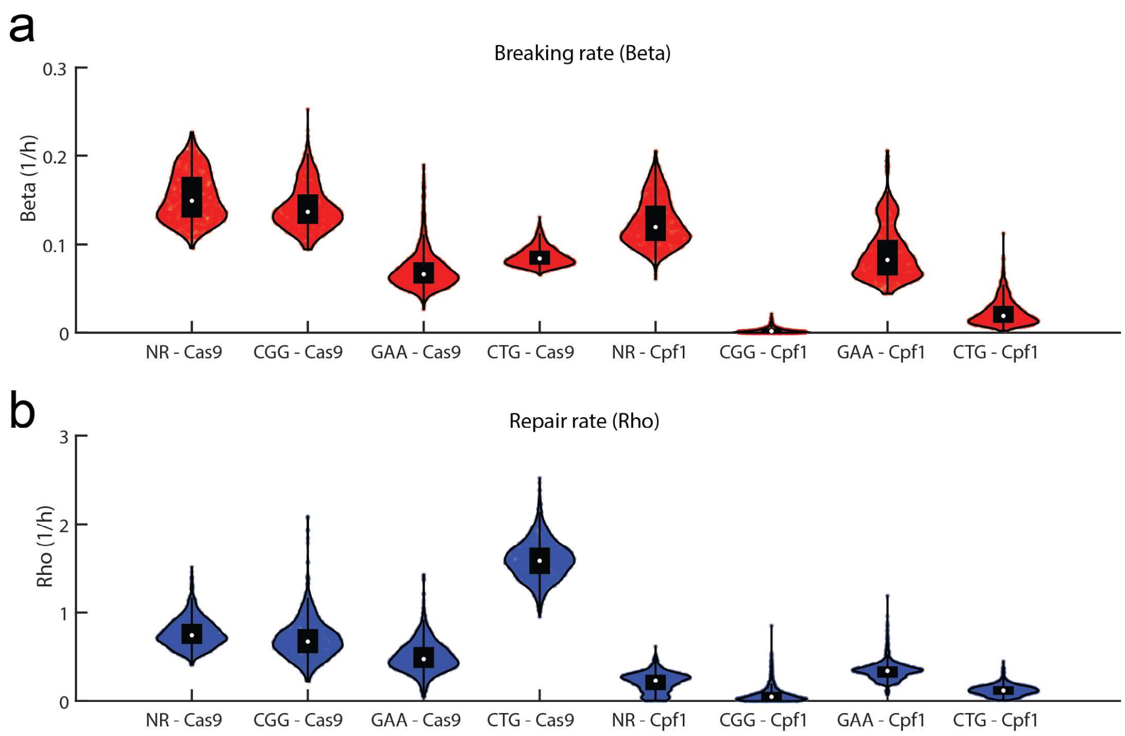


Figure S5: **Posterior distributions of breaking ($\beta$) and repair ($\rho$) rates for all experimental conditions.** **(a)** Violin plots for $\beta$ in all the conditions and **(b)** violin plots for the distribution of $\rho$. The violin plots represent the values for 1500 simulated parameter sets.

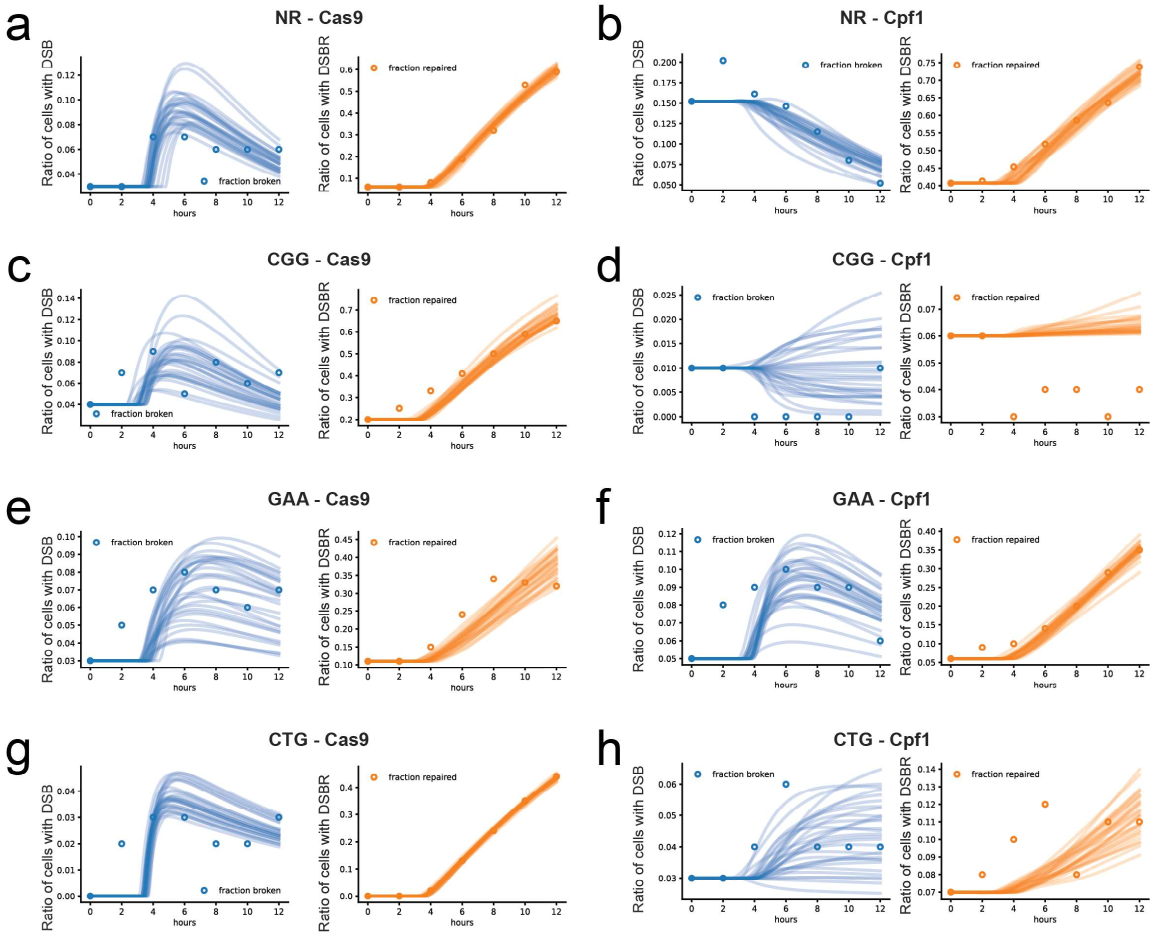


Figure S6: **Comparison of ODE model predictions to Southern blot quantification of DSB and DSBR for all experimental conditions. (a)** NR - Cas9. **(b)** NR - Cpf1. **(c)** CGG - Cas9. **(d)** CGG - Cpf1. **(e)** GAA - Cas9. **(f)** GAA - Cpf1. **(g)** CTG - Cas9. **(h)** CTG - Cpf1. In all cases, DSB is represented in blue, while DSBR is represented with red. In each plot, dots represent the Southern blot quantification and the lines the ODE simulations. Graphs show a representative sample of 20 curves taken from the full simulated data set of 1500 model parameter sets.

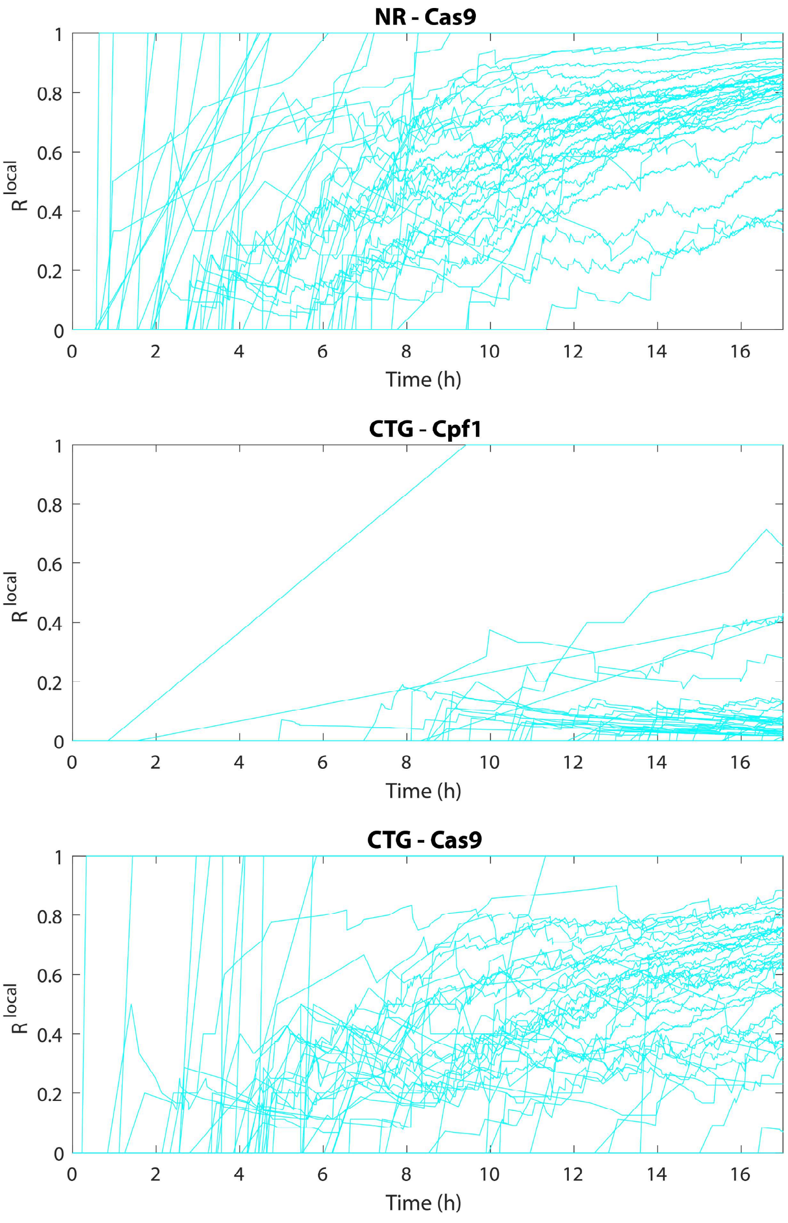


Figure S7: **Examples of individual $R^{\text{local}}$ trajectories from stochastic simulations.** NR - Cas9, CTG - Cpf1 and CTG - Cas9 cases from stochastic simulations.

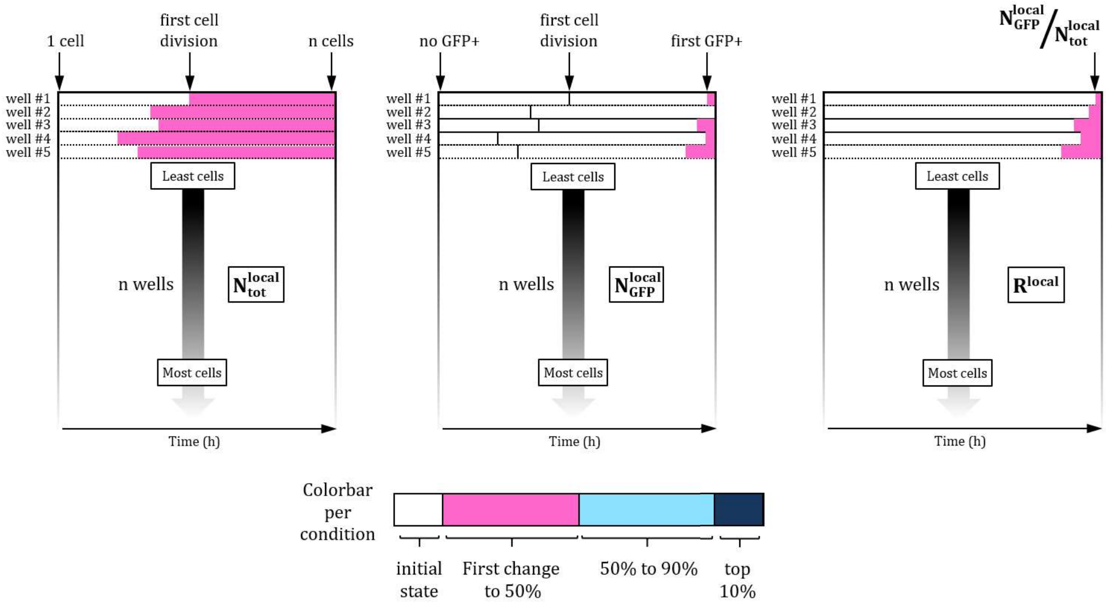


Figure S8: **Explanatory schematic for reading the heat map on Figure 6** Each row on the heat maps corresponds to data from a single well in an experiment (starting from one single cell). The rows in all three panels are sorted by the number of cells ($N_{tot}^{local}$) at $t = 24$ h, so that the top rows correspond to the lower number of cells and the bottom rows to the wells with largest number of cells at $t = 24$ h. Time goes from left to right in each panel. In the center panel the black line marks the time of the first division in that well, such that the horizontal distance between the black line and the beginning of the pink zone indicates the delay between $t_{lag}$ and $t_{GFP}$.
